# P205: An outbreak of polyclonal pseudomonas aeruginosa bacteremia in hemodialysis patients

**DOI:** 10.1186/2047-2994-2-S1-P205

**Published:** 2013-06-20

**Authors:** P Ciobotaro, A Fialko, E Nadir, M Oved, R Bardenstein, P Gershkoviz, O Zimhony

**Affiliations:** 1 Infectious Diseases Unit, Kpalan Medical Center, Aseret, Israel; 2 Harzfeld Medical Center, Gedera, Israel; 3 Infectious Diseases Unit, Kpalan Medical Center, Rehavot, Israel; 4 Infectious Diseases Unit, Harzfeld Medical Center, Gedera, Israel

## Introduction

Dialysis patients are at increased risk for infections. We describe an outbreak of *Pseudomonas aeruginosa* (*Pa*) bacteremia in patients with permcath in a dialysis unit of a 150 beds long term care facility (LTCF).

## Objectives

Epidemiological and bacteriological investigation was conducted to identify patients' risk factors, source of infection and modes of transmission.

## Methods

**Epidemiological investigation** included a plot of outbreak's curve, observations on working processes, tabulate data of possible exposures**. Bacteriologic & Molecular studies** included cultivation of dialysis-related fluids and medications, health care workers (HCW) hands and patients' surroundings. Positive cultures were analyzed for antibiotic susceptibility and clonality using random amplification of polymorphic DNA (RAPD).

In the intervention recommendations for correct working processes and training were provided to the HCW and were followed by monitoring of compliance & feedback.

## Results

18 events of *Pa* bacteremia were recorded in 12 patients who undergo hemodialysis via a permcath. 9 patients were from the same ward in the LTCF.No cases were recorded in patients with A-V fistula. All fluids and intravenous medications cultures were negative for *Pa*.

*Pa* strains were isolated from few HCW hands, one permcath dressing, shower head, patients' bathroom floor, bathroom chair and treatment cart. RAPD analysis revealed polyclonality of the strains with few matches. One bacteremic strain was identical to the strain from the same patient's bathroom shower head.

Inspections on work processes revealed that the permacath dressings got wet during bathing and were not changed until the next dialysis. Intervention that focused on proper permcath care resulted in marked decrease in incidence of *Pa* bacteremia for the following months.

## Conclusion

The polyclonality of the isolated strains combined with the matches found between bacteremic and environmental strains suggested that *Pa* strains got into permcath tunnels through an improper handling of the catheters dressing rather than due to a common infected external source. Improper catheter care resulting in wet dressings is the plausible cause. Adherence to instructions for catheter care can minimize this risk.

## Disclosure of interest

None declared

